# Relationship between vitamin D status and immunosuppressive therapy in kidney transplant recipients

**DOI:** 10.1080/13102818.2014.995415

**Published:** 2015-01-08

**Authors:** Jean Jeanov Filipov, Borelli Kirilov Zlatkov, Emil Paskalev Dimitrov, Dobrin Svinarov

**Affiliations:** ^a^ Department of Nephrology and Transplantation, University Hospital ‘Alexandrovska’, Medical University of Sofia, Sofia, Bulgaria; ^b^ Laboratory of Therapeutic Drug Management & Clinical Pharmacology, University Hospital ‘Alexandrovska’, Medical University of Sofia, Sofia, Bulgaria

**Keywords:** immunosuppressive agents, calcineurin inhibitors, renal transplantation, 25-hydroxyvitamin D

## Abstract

There is a growing body of evidence for the protective role of vitamin D in diabetes mellitus (DM), infection, cancer, cardiovascular disease, immune disorders and kidney function. Considering the reported high prevalence of vitamin D insufficiency among kidney transplant recipients (KTRs), the aim of this study was to assess the influence of immunosuppressive therapy and other factors on vitamin D status in such patients. The study included 289 KTRs (189 males and 100 females) who consented to participate. The first test for 25-hydrohyvitamin D [25(OH)D] was performed by a validated liquid chromatography–tandem mass spectrometry method. Influence of immunosuppressive drugs and previously reported predictors on vitamin D status was assessed by descriptive statistics, univariate and multivariate regression. Our results showed that only 53 patients (18.34%) of the studied KTRs were vitamin D sufficient. In addition to a well expected positive association between serum 25(OH)D and summer blood sampling (*p* < 0.05) and inverse relationship between vitamin D status and DM, gender (female) and body mass index, serum 25(OH)D was found to be inversely associated with calcineurin inhibitors (CNI) (*p* < 0.05) and unaffected by other immunosuppressive agents. Our study demonstrated high prevalence of vitamin D insufficiency after kidney transplantation in the studied cohort of patients. Apart from female gender, winter months, DM and overweight, the use of CNI could be considered an additional significant predictor of lower 25(OH)D in Bulgarian KTRs.

## Introduction

Vitamin D is an established factor in calcium–phosphorus metabolism for decades, and a new role beyond bone and mineral health is becoming evident: vitamin D is associated with glycemic control in diabetes mellitus (DM), infection, autoimmunity, renoprotection, cancer prevention and some degenerative disorders.[1] Kidney transplant recipients (KTRs) are at increased risk for DM, infection and neoplasia due to the immunosuppressive regimen.[2] Stavroulopoulos et al. [3] discovered high incidence of vitamin D insufficiency after kidney transplantation. Falkiewicz et al. [4] demonstrated that low 1,25 dihydroxyvitamin D [1,25(OH)_2_D] levels after kidney transplantation are associated with poorer outcome, with significantly increased incidence of delayed graft function, cancer and death. Furthermore, improved survival of heart, liver and kidney allografts in animal models was reported, with the use of 1,25(OH)_2_D.[5] The recognized risk factors for low vitamin D levels are race, age, seasonal variations, prevalence of chronic kidney disease (CKD), advanced liver disease, etc.[6] In KTRs, the risk of vitamin D insufficiency is further increased due to the reduced exposure to direct sunlight in order to reduce the skin cancer incidence and the use of steroids.[7]

The aim of this study was to assess the influence of immunosuppressive therapy and other factors on vitamin D status. The marker chosen was 25-hydroxyvitamin D [25(OH)D], as a generally accepted one [8].

## Subjects and methods

### Subjects

Initially, 395 patients in our transplant centre were tested for 25(OH)D for the first time after the transplantation, between 1 May 2012 and 30 November 2012. For the purpose of this study, the following selection (inclusion/exclusion) criteria were applied: KTRs less than 6 months after kidney transplantation were excluded; patients with performed parathyroidectomy and unstable kidney function were also excluded from the study; subjects with active/advanced liver disease (Child–Pugh score B and over) and with vitamin D supplementation were not taken into consideration, as well as outliers for parathyroid hormone (PTH), body mass index (BMI) and 25(OH)D (absolute value for Z-score greater than 3.29). Based on these selection criteria, 289 subjects were included in our study.

The study was approved by the Institutional Ethics Committee and was in accordance with the Helsinki Declaration of 1975 (as revised in 2000). All participants gave their informed consent prior to inclusion in the study.

### Possible predictors of vitamin D status

The following groups of factors known to affect vitamin D status were assessed: demographic (gender, age, BMI), seasonal [month of testing for 25(OH)D], metabolic [calcium, phosphorus, albumin, PTH, creatinine and estimated glomerular filtration rate (GFR) calculated according to the CKD-EPI (Chronic Kidney Disease Epidemiology Collaboration) formula]. Several diseases known for their influence on 25(OH)D were evaluated: DM, cancer and rejection episode within 12 months from 25(OH)D testing. Different immunosuppressive medications (steroids, pulse steroids within 12 months from 25(OH)D testing, mycophenolic acid derivatives, azathioprine, cyclosporin A (CsA), tacrolimus (Tac) and mTOR (mammalian target of rapamycin) inhibitors (mTORI) were also assessed. Additional factors of consideration were time (in months) after kidney transplantation, presence of viral hepatitis and urinary tract infections.

### Analysis of 25(OH)D

Determination of 25(OH)D was performed by a validated liquid chromatography–tandem mass spectrometry (LC-MS/MS) method developed in-house, utilizing extraction with hexane, d325(OH)D3 as internal standard, isocratic elution in a C18 analytical column, positive-ion electrospray ionization and selected reaction monitoring for the respective *m*/*z* transitions: 401→383 for 25(OH)D3, 413→395 for 25(OH)D2 and 404→386 for d325(OH)D3. The method was calibrated with the use of commercial, NIST (National Institute of Standards and Technology, USA) Standard Reference Material (SRM) 972 and was validated according to FDA (Food and Drug Administration, USA) guidance requirements, with documented selectivity and matrix effect, accuracy and precision within 7.5%; extraction recoveries averaging 57%–73%; linearity range 3.0–300.0 nmol/L, *R* ^2^ > 0.99, freeze–thaw stability for three cycles of 24 h, post-preparative stability for 96 h at 10 °C, short-term stability at ambient temperature for 24 h in the dark and for 2 h at daylight; stock solution stability and long-term stability in plasma for 5 days at 4–8 °C, and for 99 days at −20 °C. It participated in DEQAS (UK Vitamin D External Quality Assessment Scheme) external proficiency testing scheme with obtained certification for 2012.

### Analysis of immunosuppressive drugs (cyclosporine, tacrolimus, sirolimus and everolimus) in human whole blood

Determination of CsA, Tac, sirolimus (SRL) and everolimus (EVR) was performed by a developed and validated in-house LC-MS/MS method utilizing methanol/Zn sulphate solution for cell lysis, extraction with 1-chlorobutane, [D12]CsA (dCsA), ascomycin (Asco) and [13c2d4]RAD001 (cdRAD) as internal standards, isocratic elution in a C18 analytical column, positive-ion electrospray ionization, and selected reaction monitoring for the respective *m*/*z* transitions: 1203→425 for CsA, 822→766 for Tac, 932→865 for SRL, 976→909 for EVR, 1215→437 for dCsA, 809→756 for Asco and 982→915 for cdRAD. The method was validated according to FDA guidance requirements, with documented selectivity and matrix effect, accuracy and precision within 8.9%; extraction recoveries averaging 65%–76%; linearity range with *R* ^2^ > 0.99, freeze–thaw stability for three cycles of 24 h, post-preparative stability for 96 h at 10 °C, short-term stability at ambient temperature for 12 h in the dark and for 6 h at daylight; stock solution stability and long-term stability in whole human blood for 7 days at 4–8 °C, and for 120 days at −20 °C. It participated in ASI (UK Analytical Services International Proficiency Testing Scheme) external proficiency testing scheme with obtained certification for 2002 until present.

Serum creatinine, calcium, phosphates, alkaline phosphatase and other routine laboratory tests were performed on a standard clinical chemistry analyser.

### Statistical analysis

Descriptive statistics, stepwise multivariate log–linear regression were used to investigate the association between 25(OH)D and explanatory variables. Spearman correlation coefficients were used to express associations between continuous parameters. The *P* < 0.05 level of significance was adopted. SPSS 22.0 (Statistical Package for the Social Sciences) Software (SPSS Inc., Chicago, IL, USA) was used.

To address the residuals heteroscedasticity, natural logarithm of the quantitative values for BMI, 25(OH)D levels, PTH and alkaline phosphatase was used.

In order to avoid distortions of parameter and statistic estimates, we screened the data for BMI, PTH and 25(OH)D level for outliers, using the Z-score method, with cut-off values lower than /−3.29/ and higher than /+3.29/.

## Results and discussion

### Patients’ characteristics

According to the predefined selection criteria, the study encompassed 289 subjects; all were Caucasians; males were predominant. The basic characteristics of the study subjects are summarized in greater detail in Table 1.

**Table 1.** Basic characteristics of the kidney transplant recipients included in the study.

	Males	Females	Total
*n*	189	100	289
Age (years)	42.80 ± 13.12	42.47 ± 11.59	42.69 ± 12.59
Time after TR* (months)	92.84 ± 61.42	95.61 ± 63.79	93.80 ± 62.16
eGFR^†^ (mL/min/1.73 m^2^)	63.11 ± 21.37	65.40 ± 26.36	63.90 ± 23.19
BMI^§^	24.86 ± 4.79	23.73 ± 4.79	24.44 ± 4.38
Vitamin D concentration^‡^ (nmol/L)	63.97 ± 23.31	49.73 ± 21.02	59.04 ± 23.51

*TR – transplantation, ^†^eGFR – estimated glomerular filtration rate (CKD-EPI formula), ^§^BMI – body mass index, ^‡^ total 25(OH)D.

**Figure 1.**
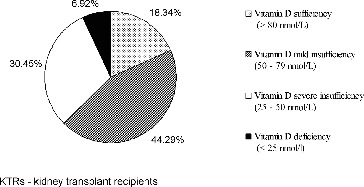
Vitamin D status of the studied kidney transplant recipients (*n* = 289).

High prevalence of suboptimal vitamin D status was detected, with more than 80% of patients having 25(OH)D concentrations below 80 nmol/L. Mild insufficiency was the most common abnormality. The results are shown in Figure 1.

**Figure 2.**
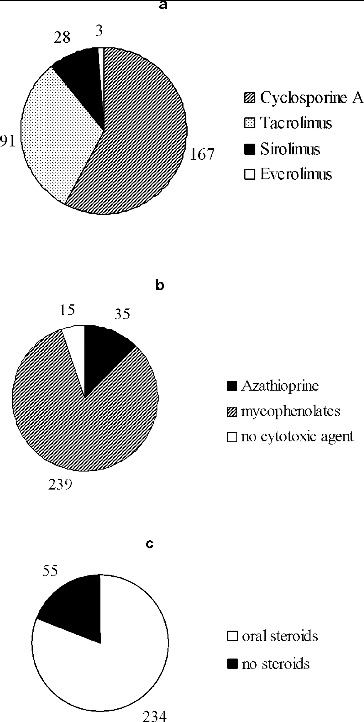
Immunosuppressive agents in the studied cohort of patients (*n* = 289). CNIs and mTORI (**a**); cytotoxic agents (**b**); oral steroids (**c**).

Most of the KTRs were on triple immunosuppressive regimen (CNI, calcineurin inhibitors, or mTORI plus azathioprine or mycophenolate plus steroids). Details about the immunosuppressive agents are given in Figure 2.

### Factors affecting vitamin D status

Spearman correlation analysis revealed a significant negative association between vitamin D status and BMI and a significant positive one between vitamin D and time after transplantation. There was insignificant correlation between vitamin D and the following continuous parameters: proteinuria, age of KTRs, CsA and Tac level, calcium and phosphorus serum level, estimated GFR and proteinuria (Table 2).

**Table 2.** Spearman correlation analysis of 25(OH)D serum level and continuous parameters in kidney transplant recipients (*n* = 289).

Parameter	Correlation coefficient (*r*)
Age (years)	−0.071
Time after transplantation (months)	0.126*
BMI	−0.130*
Ca level	0.075
P level	0.003
CsA level	−0.057
Tac level	−0.074
eGFR	0.075
Proteinuria (g/24 h)	−0.024

*Correlation is significant at the 0.05 level (2-tailed).

BMI – body mass index, Ca – serum calcium, P – serum phosphorus, CsA – Cyclosporin A, Tac – tacrolimus, eGFR – estimated glomerular filtration rate.

Due to the large number of possible factors influencing vitamin D status in KTRs, stepwise multivariate analysis was performed. Among all factors tested, several statistically significant predictors were identified, with negative or positive influence on 25(OH)D values (Table 3). As expected, there was negative association between the concentration of 25(OH)D and female gender, presence of DM and BMI. In addition, CNI intake was also found to negatively affect 25(OH)D. The results confirmed a positive seasonal influence on 25(OH)D (blood sampling in summer months). All other tested factors gave insignificant results in regard to influence on the vitamin D status, including other immunosuppressive medications such as oral steroids, pulse steroids within 12 months prior to vitamin D testing, azathioprine and mycophenolates. In contrast to CNI, mTORI (both EVR and SRL) also did not affect 25(OH)D (the small number of subjects on EVR – only 3 – should be noted here). Again, some well-known factors affecting vitamin D status, such as age, renal function and proteinuria, were found to be insignificant in our study.

**Table 3.** Factors influencing 25-hydroxyvitamin D levels.

	β	SE	*P* value
Intercept	5.994	0.621	<0.0001
Females	−0.332	0.052	<0.0001
July	0.254	0.101	0.012
August	0.444	0.084	<0.0001
September	0.329	0.079	<0.0001
Diabetes mellitus	−0.181	0.091	0.048
ln BMI	−0.900	0.361	0.013
CNI	−0.177	0.084	0.036
Age (years)	0.0001	0.002	0.963
Proteinuria (g/L)	−0.028	0.028	0.325
ln eGFR	−0.019	0.071	0.791
Oral steroids	0.015	0.066	0.821
Pulse steroids	−0.096	0.086	0.264
Azathioprine	−0.159	0.147	0.279
Mycophenolates	−0.099	0.126	0.435
mTORI	0.124	0.084	0.144

Dependent variable: ln 25-hydroxyvitamin D; adjusted *R* ^2^ = 0.345.

BMI – body mass index, SE – standard error; eGFR – estimated glomerular filtration rate (CKD-EPI), CNI – calcineurin inhibitors, mTORI – mammalian target of Rapamycin inhibitors.

### Final remarks

Considering the multiple effects of vitamin D, it is of utmost importance to detect the factors that influence its status in a particular patient population. In this study, we discovered high prevalence of vitamin D insufficiency after kidney transplantation, similar to the findings of other authors [3]. In addition, we report several predictors of 25(OH)D concentrations in KTRs. The factor with the highest influence was female gender, indicating that females are at higher risk for low vitamin D, a result which is in accordance with the data of Mithal et al [9]. Summer blood sampling, BMI and DM have already been recognized as predictors for 25(OH)D concentrations.[10] Marked seasonal influence was also detected in our patients. Although the seasonal nadir was not encompassed in the study, vitamin D deficiency and insufficiency were still widespread in more than 80% of the patients. We may expect that, in winter, the inadequacy rate will increase further, thus making vitamin D deficiency a much more serious problem for the KTRs. Our results clearly showed that higher BMI and presence of DM affect negatively 25(OH)D. Low 25(OH)D concentrations in obese patients could partly be explained with sequestration of vitamin D in the adipose tissue. Although DM has a negative effect on serum 25(OH)D, improvement in glycemic control only weakly improves vitamin D status in diabetics.[11]

The interaction between immunosuppressive medications and vitamin D has been studied extensively. So far steroids have been recognized as a factor for lower 25(OH)D. These drugs increase 24-hydroxylase activity through activation of pregnane X receptor.[10] The relationship between CNIs and vitamin D metabolism has been studied with conflicting reports. Grenet et al. [12] reported increased 1,25-(OH)_2_D levels, decreased calbindin-D28k, decreased vitamin D receptor and 24-hydroxylase expression in Wistar rats treated with CsA. Another group established no influence of CsA on vitamin D concentrations in patients with multiple sclerosis.[13] Our results indicate that CNI intake is associated with lower 25(OH)D concentrations, while treatment with mTORI does not affect vitamin D status after renal transplantation. These effects appear to be drug-class but not drug-concentration dependent. Lee et al. [14] demonstrated that CNIs, but not SRL, induce vitamin D resistance. Eyal et al. [15] found negative influence of Tac and other immunosuppressive medications on 25(OH)D in KTRs. A possible explanation for these findings may be the fact that liver CYP3A4 has 25-hydroxylase activity which is suppressed by CsA and TAC resulting in lower 25(OH)D.[16] All other immunosuppressive agents, including oral steroids, had a non-significant effect on 25(OH)D level in our cohort of subjects.

Our study was performed retrospectively, which is its major limitation. A prospectively designed research would further be needed for a more accurate assessment of the link between CNI and vitamin D status.

## Conclusions

Our study established high prevalence of suboptimal levels of 25(OH)D in KTRs. The vitamin D status of the patients in our transplant centre was influenced by a broad spectrum of factors. In addition to the well-known determinants of 25(OH)D (seasonal variations, DM, obesity, gender), significant influence of CNI intake on vitamin D was observed. As CNIs are currently the backbone of immunosuppressive treatment after renal transplantation, further large-scale prospective studies are obviously needed to explicitly clarify the possible link between immunosuppressive therapy and vitamin D in KTRs.
